# Metabolomics investigation of the chemical variations in white teas with different producing areas and storage durations

**DOI:** 10.1016/j.fochx.2024.101127

**Published:** 2024-01-06

**Authors:** Zewen Chen, Weidong Dai, Mengfan Xiong, Jianjian Gao, Hongjie Zhou, Dan Chen, Yali Li

**Affiliations:** a College of Tea, Yunnan Agricultural University, Kunming, Yunnan, 650201, China; b Tea Research Institute, Chinese Academy of Agricultural Sciences, Hangzhou, Zhejiang 310008, China

**Keywords:** Metabolomics, Producing areas, Storage, White tea, *N*-ethyl-2-pyrrolidone-substituted flavanols

## Abstract

In this study, we employed nontargeted metabolomics and quantitative analysis to explore the variations in metabolites among white teas from different production areas and with varying storage durations. A total of 83 compounds exhibited differential levels between Zhenghe and Fuding white tea, 89 between Zhenghe and Jinggu, and 75 between Fuding and Jinggu white tea. Concerning the storage of white tea, the concentrations of flavanols, dimeric catechins, and amino acids decreased over time, while *N*-ethyl-2-pyrrolidone-substituted flavanols (EPSFs), caffeine, adenosine monophosphate (AMP), and adenosine increased. Galloylated flavanols showed a higher propensity to form EPSFs with theanine compared to nongalloylated flavanols during storage. Theanine and epigallocatechin gallate were more inclined to generate S-configuration EPSFs during storage in Fuding and Jinggu white tea samples, while R-configuration EPSFs were more readily formed in Zhenghe white tea samples. This study offers a comprehensive understanding of the changes in metabolites during the storage of white tea.

## Highlights

• Differential compounds were identified from three different habitats.
• Amino acids and flavonols had the largest number of differences.
• R-configuration EPSF were more easily formed in ZHWT samples, FDWT and JGWT were S-configuration.
• The content of aroma precursors in ZHWT was much highest.

## 1 Introduction

Tea has being widely consumed and recognized as a health-promoting beverage. There are various types of tea, such as white, yellow, green, oolong, black, and dark teas (Feng et al., 2019, Jiang et al., 2022). White tea, a traditional Chinese tea, undergoes a simple process involving withering and drying of fresh tea leaves, resulting in a sweet-mellow taste and fresh aroma due to chemical reactions among aroma glycoside precursors, amino acids, tea polyphenols, and other substances (Chen et al., 2023, Deng et al., 2022, Zhou et al., 2023). Its exceptional health benefits, attributed to high contents of polyphenols, amino acids, alkaloids, and other beneficial compounds, distinguish white tea. These components have been associated with anti-cancer (David et al., 2021), anti-diabetic (Xia et al., 2020), anti-inflammatory (Berilli et al., 2022), antioxidant (Pérez-Burillo, Giménez, Rufián-Henares, & Pastoriza, 2018), and various other health-promoting properties (Fan et al., 2021, Zhang et al., 2019).White teas are typically categorized into four grades based on the freshness and tenderness of the tea leaves, ranging from the highest to the lowest quality. These grades include Baihaoyinzhen (also known as Silver Needle, crafted from tea buds), Baimudan (or White Peony, made from a bud with one or two leaves), Gongmei (crafted from a bud with more than two leaves), and Shoumei (produced from young shoots and more than two leaves) (Huang et al., 2021). Among these, Baimudan, currently the most popular white tea, is further classified into four grades, each offering a distinct aroma and flavor profile, with distinctions made between super, grade I, grade II, and grade III (Yue et al., 2019). In general, China has a diverse range of white teas, each region producing tea with unique characteristics. Fujian province, renowned for its white tea production, features Fuding County and Zhenghe County as its most prominent production areas. White tea from Fuding and Zhenghe is favored for its gentle and mellow taste. In recent years, the growing popularity of white tea has led to production in other regions, such as Yunnan, where Jinggu white tea has gained recognition for its exceptional taste and quality.

There's a Chinese proverb that describes white tea as “one-year tea, three-year medicine, seven-year treasure” indicating that storage significantly influences the taste, aroma, chemical composition, and health benefits of white tea. Moreover, stored white tea often holds higher commercial value (Wang et al., 2023, Wang et al., 2023). During the storage period, notable changes occur: herbal and sweet aromas become more pronounced while green and grassy notes diminish (Qi et al., 2018). Simultaneously, the bitterness and astringency decrease, while thickness, acidity, sweetness, and smoothness increase (Fan et al., 2021). Furthermore, long-term storage results in a reduction in non-volatile compounds, including most amino acids, catechins, flavonol/flavone glycosides, dimeric catechins, and lipids. In contrast, there is an increase in *N*-ethyl-2-pyrrolidinone-substituted flavan-3-ols (Dai et al., 2018, Xie et al., 2019). Additionally, the duration of storage also impacts the biological activities of white tea, with antioxidant properties and inhibitory effects on *α*-glucosidase and *α*-amylase diminishing as the storage period extends (Xu, Chen, & Wang, 2019).

The quality of white tea is subject to variations based on its origin and storage duration. Nonetheless, the relationship between the chemical components of white tea and its origin and storage duration remains unclear. As the application of metabolomics in the study of tea quality chemistry becomes increasingly widespread and enables the quantification of a plethora of compounds in tea (Shan et al., 2023, Wang et al., 2023, Wang et al., 2023, Zhou et al., 2022), the need for a high-throughput method to identify compounds associated with the tea's origin and storage duration has arisen. Therefore, this study utilizes a metabolomics approach, employing an ultra-high-performance liquid chromatography-quadrupole/electrostatic field orbitrap mass spectrometer (UHPLC-Q-Exactive/MS), to analyze Baimudan grade I samples from diverse origins and varying storage durations, aming to unveil the links between the chemical components in white tea, its origin, and the duration of storage.

## 2 Materials and methods

### 2.1 Chemicals and regents

Dimeric catechins, such as theaflavin-3ʹ-gallate (TF-3ʹ-G), theaflavin-3,3ʹ-digallate (TF-DG), theaflavin-3-gallate (TF-3-G), theaflavin (TF), procyanidin B2, and procyanidin B1, were sourced from ChemFaces (Wuhan, Hubei, China). Additionally, the following compounds were procured from Sigma (St. Louis, MO, USA): nine flavanols, namely (−)-epigallocatechin (EGC), (−)-epicatechin (EC), (−)-gallocatechin gallate (GCG), (−)-epigallocatechin gallate (EGCG), (+)-catechin (C), (−)-gallocatechin (GC), (−)-epicatechin gallate (ECG), (−)-epigallocatechin-3-(4′'-O-methyl) gallate (EGCG-4′'-O-Me), and (−)-epiafzelechin 3-gallate; fifteen amino acids; eight kaempferol-O-glycosides and quercetin-O-glycosides; as well as flavone-C-glycosides, including vitexin and isovitexin. Other chemicals such as 1-ethyl-5-hydroxy-2-pyrrolidinone, quinic acid, benzyl primeveroside, pyroglutamic acid, pipecolic acid, choline, caffeine, and adenosine monophosphate (AMP), were procured from Yuanye Bio-Technology Co., Ltd. (Shanghai, China). Milli-Q water was used in this study (Millipore, Billerica, MA, USA). Liquid chromatography-mass spectrometry (LC-MS) grade formic acid, methanol, and acetonitrile of were obtained from Merck (Darmstadt, Germany) (Gao et al., 2022, Gao et al., 2022, Peng et al., 2021).

### 2.2 Sample collection

In our previous studies, we found that the compounds changed more obviously at the early stage of storage than at the late stage of storage (Dai et al., 2020, Chen et al., 2021). In this study, the samples of Zhenghe white tea (ZHWT), Fuding white tea (FDWT), and Jinggu white tea (JGWT) were collected from different production years, including 2022, 2021, 2020, 2019, and 2007 that with storage years of 0, 1, 2, 3, and 15 years, respectively. These tea samples have dense sampling points (0, 1, 2, and 3 years) at the early stage of storage to investigate the compound variation in detail. ZHWT and FDWT samples were collected from Zhenghe (Fujian province, China) and Fuding (Fujian province, China), respectively, and they are representative Fujian white teas; JGWT was collected from Jinggu (Yunnan province, China) and is a representative Yunnan white tea.These three kinds of white tea occupy a large part of the white tea market in China.

Their growth environment and processing technology are different. FDWT growth in the eastern subtropical Marine monsoon climate area, mild and humid climate, abundant rainfall, the soil is mainly medium and light soil; ZHWT is a subtropical monsoon humid climate zone, with four distinct seasons, rainy spring and summer, and the soil is mainly red loess. JGWT grows in the subtropical mountain monsoon climate area, high temperature and rainy in summer, cold and little rain in winter, the soil is mainly red soil.

In this experiment, the processing technology of FDWT is mainly to dry the fresh leaves after picking, and then to bake at low temperature or dry at low temperature. ZHWT usually wilts naturally indoors and then is dried by low-temperature baking or sunlight; JGWT withers indoors throughout the whole process and does not need to be baked.

All the samples were of Baimudan grade I, consisting of one bud and one leaf. The tea leaves were meticulously ground into a fine powder using an IKA mill (Staufen, Germany). The sensory evaluation of the tea samples followed the guidelines outlined in *GB/T23776-2018* '*Tea sensory evaluation method*'. This evaluation involved five experienced tea technicians who were invited to assess the tea samples based on various criteria, including their appearance, liquor color, aroma, taste, and the characteristics of the infused leaves.

### 2.3 Extraction of chemical constituents from white tea samples

Precisely weighted 0.1 g of tea powder sample was transferred into a 50 mL centrifuge tube and a 15 mL of 70 % methanol solution was added, then the tube was thoroughly mixed by vigorous shaking. Subsequently, the centrifuge tube was incubated in a 70 °C water bath for 30 min, intermittently shaking every 5 min. The solution was centrifuged at 9000 *g* for 10 min. Then the supernatants was filtered through a 0.22-μm nylon filter membrane, and injected into LC-MS for quantitative measurement and metabolomics analysis. Three technical replicates were performed for each tea sample (Dai et al., 2020).

### 2.4 LC-MS conditions for metabolomics analysis

The untargeted metabolomics analysis of the white tea samples was performed in accordance with established protocols from our prior research (Chen et al., 2022, Sun et al., 2022). Data acquisition was carried out using a UHPLC-Q-Exactive/MS instrument from Thermo Fisher Scientific (Waltham, MA, USA). An Acquity UPLC HSS T3 column (2.1 mm × 100 mm, 1.8 μm, Waters, UK) was utilized. The instrumental LC-MS settings were consistent with those employed in our earlier studies (Chen et al., 2022, Sun et al., 2022). The gradient elution was as follows: 0 min, 98 % A (water with 0.1 % formic acid) and 2 % B (acetonitrile with 0.1 % formic acid); 0.5 min, 2 % B; 10 min, 15 % B; 18 min, 40 % B; 20 min, 90 % B; 20.9 min, 90 % B; 21 min, 2 % B; and 25 min, 2 % B. The flow rate was 0.4 mL/min and run time was 25 min for injection. The injection volume was 3 μL. The data were acquired in positive electrospray ionization (ESI^+^) mode, other parameters settings were as follows: mass range of *m*/*z* 80–1200, the capillary temperature was 300 °C, capillary voltage was set at 3.5 kV, the sheath and auxiliary gas flow was 40 and 10 arb in positive ionization mode, respectively. The temperature of drying gas was 350 °C and the flow rate of the drying gas maintained at 10 L/min. Additionally, equivalent amounts of each white tea sample were employed to generate quality control (QC) samples, which were included in the analytical sequence to ensure data quality.

### 2.5 Quantitative analysis of the EPSFs in stored white teas

The quantification of EPSF compounds in white tea samples was carried out using the external standard method. This involved quantifying the following compounds: 5″ S-epicatechin-C *N*-ethyl-2-pyrrolidinone (S-EC-cThea), 5″ R-epicatechin-C *N*-ethyl-2-pyrrolidinone (R-EC-cThea), 5″ S-epigallocatechin-C *N*-ethyl-2-pyrrolidinone (S-EGC-cThea), 5″ R-epigallocatechin-C *N*-ethyl-2-pyrrolidinone (R-EGC-cThea), 5‴ S-epicatechin gallate-C *N*-ethyl-2-pyrrolidinone (S-ECG-cThea), 5‴ R-epicatechin gallate-C *N*-ethyl-2-pyrrolidinone (R-ECG-cThea), 5‴ S-epigallocatechin gallate-C *N*-ethyl-2-pyrrolidinone (S-EGCG-cThea), and 5‴ R-epigallocatechin gallate-8-C *N*-ethyl-2-pyrrolidinone (8-C R-EGCG-cThea) by calibration curve of 8-C R-EGCG-cThea. The calibration curve for 8-C R-EGCG-cThea was established using standard solutions of 0.01, 0.05, 0.1, 0.2, 0.5, and 1.0 mg/mL.

### 2.6 Data processing, analysis and statistics

The peak picking and alignment of the LC-MS raw data were processed by Compound Discoverer 3.2 software (Thermo Fisher Scientific; Waltham, MA, USA). The retention time width was 0.2 min and the mass width was set at 10 ppm.

The supervised partial least squares discriminant analysis (PLS-DA), loading plot, and permutation test were performed by SIMCA-P 14.1 software (Umetrics AB, Umeå, Sweden). Heatmap analysis was performed using MultiExperiment Viewer software (version 4.7.4, Oracle, USA). Student’s *t*-test and Duncan’s test were performed using SPSS 25 (IBM, Chicago, IL, USA).

## 3 Results and discussion

### 3.1 Sensory quality of white teas of different storage years

The sensory quality of ZHWT, FDWT and JGWT samples was evaluated in terms of aroma, taste, and color. As delineated in Table 1, the duration of storage exhibited minimal influence on the visual aspects, but notably affected the tea's aroma, liquor color, taste, and characteristics of the infused leaves. In terms of aroma and taste quality, the initial delicate aroma of white tea from the three different producing areas evolved into an herbal fragrance after undergoing storage. Furthermore, the initial taste profile, characterized by notes of sweetness, freshness, bitterness, and a slight astringency, transitioned into a sweeter and mellower taste profile. The liquor color shifted from a light apricot and wax yellow hue to a honey yellow tone, while the infused leaves transformed from a yellowish green shade to a soft, bright, and greyish auburn color. Given the significant impact of non-volatile compounds on tea quality, the changes in these compounds during storage were subjected to further analysis.

**Table 1:** Sensory evaluation of Zhenghe, Fuding, Jinggu white tea samples during storage.

Samples	Appearance	Aroma	Liquor color	Taste	Infused leaf
Z07	whole shoot, slightly tippy, even, greyish auburn	strong and lasting, herbal aroma	honey yellow	sweet and mellow, sweet after taste	greyish auburn, soft and bright
Z19	whole shoot, slightly tippy, even, greyish auburn	fragrant and lasting, fruity aroma	honey yellow	sweet and mellow, sweet after taste	greyish auburn, soft and bright
Z20	whole shoot, slightly tippy, greyish green	fruity and honey aroma, herbal aroma	apricot	sweet and mellow, bitter	yellowish green
Z21	whole shoot, slightly tippy, even, greyish green	strong and lasting, herbal aroma	apricot	sweet and fresh, bitter	yellowish green
Z22	whole shoot, slightly tippy, blueish auburn	strong and lasting, tip aroma	wax yellow	sweet and fresh, sweet after taste, bitter	yellowish green
F07	whole shoot, slightly tippy, even, greyish auburn	strong and lasting, herbal aroma	honey yellow	sweet and mellow, sweet after taste	greyish auburn, soft and bright
F19	whole shoot, slightly tippy, even, greyish green	high aroma, flowery and fruity aroma	apricot	sweet and mellow, fresh and brisk	greyish green, soft and bright
F20	whole shoot, slightly tippy, even, greyish green	strong and lasting, fruity and honey aroma	apricot	sweet, sweet after taste, slightly astringent	yellowish green, soft and bright
F21	whole shoot, slightly tippy, even, greyish green	flowery and fruity aroma	apricot	sweet and fresh, sweet after taste	yellowish green
F22	whole shoot, slightly tippy, greyish green	strong and lasting, tip aroma	light apricot	sweet, fresh and brisk, slightly astringent	yellowish green
Y07	whole shoot, slightly tippy, even, greyish auburn	strong and lasting, herbal aroma	honey yellow	sweet and mellow, sweet after taste	brownish auburn, soft and bright
Y19	whole shoot, slightly tippy, even, greyish auburn	strong and lasting, fruity and honey aroma	honey yellow	sweet and mellow, sweet after taste	brownish auburn, soft and bright
Y20	whole shoot, slightly tippy, even, greyish auburn	fragrant and lasting, flowery aroma	bright yellow	sweet and mellow, sweet after taste	fat and tender, yellowish auburn, soft and bright
Y21	whole shoot, slightly tippy, greyish auburn	strong and lasting, flowery and honey aroma, tip aroma	honey yellow	sweet and fresh, sweet after taste	yellowish auburn
Y22	whole shoot, slightly tippy, greyish auburn	flowery aroma, tip aroma	light wax yellow	sweet and fresh, sweet after taste	yellowish auburn

### 3.2 Metabolite characterization of white tea from three different producing areas

**Fig. 1:**
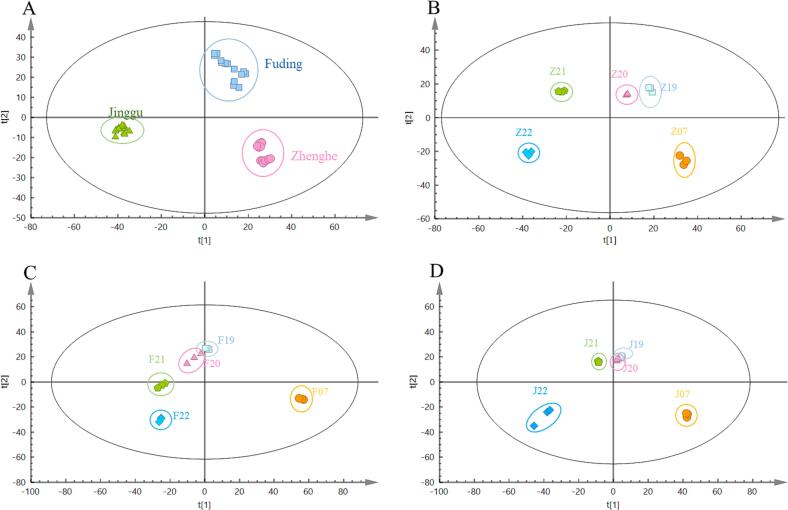
PLS-DA score plot of the compound patterns in (A) white tea from three different producing areas and in (B) Zhenghe, (C) Fuding, and (D) Jinggu white tea samples with different storage durations. Pink circles, light blue squares and green triangles represent the Zhenghe, Fuding and Jinggu white tea samples, respectively. (For interpretation of the references to color in this figure legend, the reader is referred to the web version of this article.)

We conducted an untargeted metabolomic analysis to comprehensively examine the global metabolic profiles within the white tea samples (Fig. S1-A). After peak alignment, picking, signal denoising, and data preprocessing, we retained a total of 1489 metabolite ions for analysis. PLS-DA (Fig. 1A) demonstrated distinct clustering and separation trends among JGWT samples compared to ZHWT and FDWT samples along the primary component 1 axis (R2X[1] = 0.199). Furthermore, along the primary component 2 axis (R2X[2] = 0.088), evident clustering and separation were observed, with FDWT samples distinctly separating from JGWT and ZHWT samples. These findings underscore the differences in compound contents among white tea samples from the three distinct producing areas. To ensure the model's reliability, a permutation test (n = 100) (visualized in Fig. S2-A) was executed, revealing R^2^ and Q^2^ intercepts of 0.227 and −0.23, respectively, indicating that the PLS-DA model was not overfitted and therefore was reliable. A loading plot was used (Fig. S3) to identify key metabolites responsible for distinguishing between ZHWT, FDWT, and JGWT samples. The compounds represented by orange triangles in the loading plot were identified as contributors to the classification, prompting further structural analysis and identification of these compounds.

A total of 154 non-volatile compounds were structurally identified through authentic standards verification, combined with our previous works (Chen et al., 2022, Chen et al., 2021, Dai et al., 2018) and searching in metabolomics databases (HMDB and Metlin), which included 11 flavanols (EGCG, EGC, ECG, etc.), 13 dimeric catechins (theaflavins, procyanidin C1, theasinensin A, theasinensin F, etc.), 17 amino acids (theanine, tryptophan, proline, glutamine, etc.), 14 alkaloids (caffeine, theobromine, AMP, choline, etc.), 13 phenolic acids (4-coumaroylquinic acid, 3-coumaroylquinic acid, theogallin, chlorogenic acid, etc.), 9 organic acids (quinic acid, picolinic acid, vanillic acid, isocitric acid, etc.), 15 EPSFs (8-C S-EGC-cThea, 8-C R-EC-cThea, 8-C R-EGCG-cThea, 8-C S-ECG-cThea, etc.), 8 aroma precursors (benzyl glucoside, phenylethyl primeveroside, benzyl primeveroside, geranyl primeveroside, etc.), 5 flavonols (narigenin, kaempferol, quercetin, myricetin, etc.), 26 flavonol/flavone glycosides (kaempferol-3-glucoside, quercetin-3-glucoside, myricetin 3-glucoside, apigenin-6-C-glucosyl-8-C-arabinoside, etc.), 16 lipids (palmitic acid, phytosphingosine, MG (18:0), lysoPC (16:0), etc.), 7 others (1-ethyl-5-hydroxy-2-pyrrolidinone, dihydroactinidiolide, theanine glucoside, glucose, etc.) (Table S1).

**Fig. 2:**
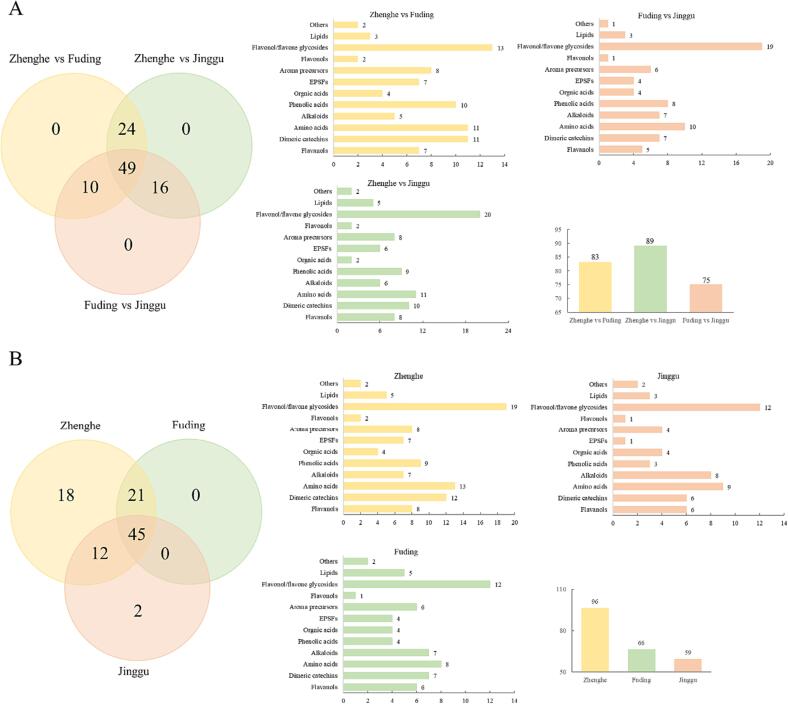
(A) The numbers of differential compounds (VIP > 1 and Duncan’s test *P*-value < 0.05) between Zhenghe-Fuding, Zhenghe-Jinggu, and Fuding-Jinggu white tea samples; (B) Differential compounds among the three different producing areas (Zhenghe, Fuding and Jinggu).

The variable importance of projection (VIP) value is an indicator of the extent to which each compound contributes to the distinctions in the PLS-DA model. A higher VIP value signifies a more significant contribution to the classification in the PLS-DA model and a more pronounced variation in compound content among different producing areas. To identify compounds with substantial variations in white teas from the three producing areas, we employed the criteria of VIP >1 and *P*-value <0.05 (Duncan's test). This analysis revealed a total of 83 (ZHWT vs. FDWT), 89 (ZHWT vs. JGWT), and 75 (FDWT vs. JGWT) differential compounds in the white tea samples from the three distinct producing areas (as illustrated in Fig. 2A). Notably, amino acids and flavonol/flavone glycosides exhibited the highest number of disparities. These two compound categories accounted for a significant proportion of the identified differences, ranging from 12.4 % to 13.3 % for amino acids and 15.7 % to 25.3 % for flavonol/flavone glycosides, surpassing the other compound groups.

**Fig. 3:**
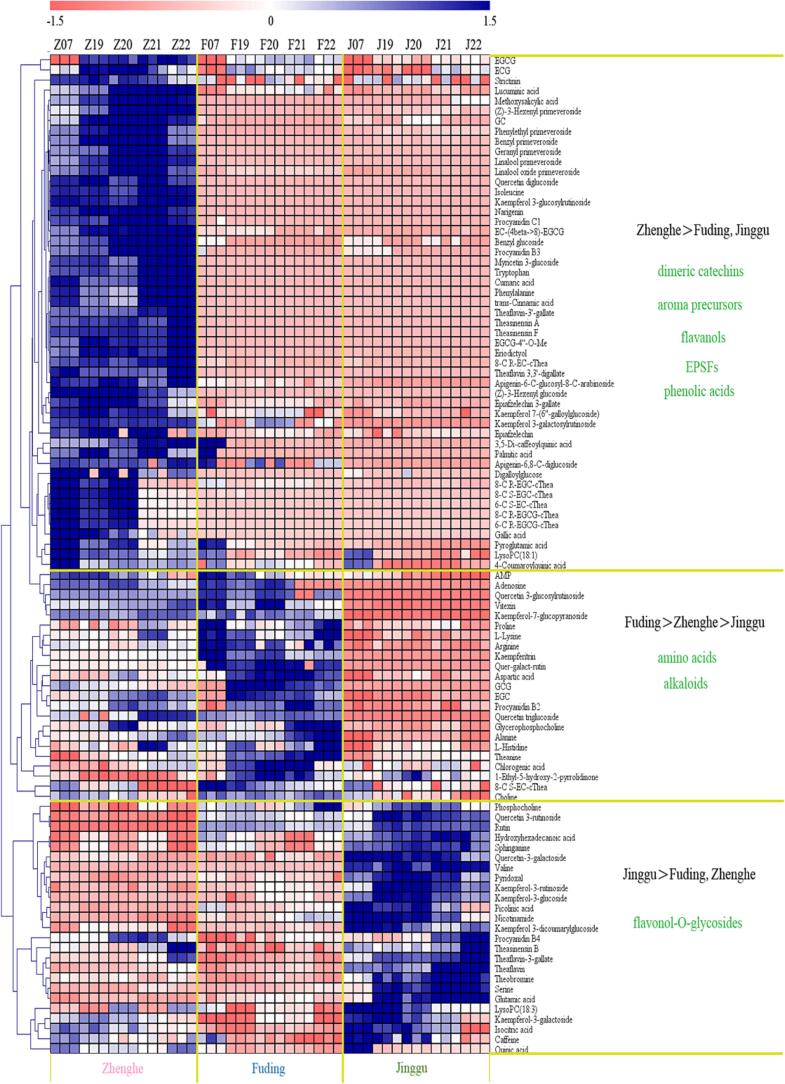
Heatmap analysis of the contents of 99 producing area-related differential compounds in Zhenghe, Fuding, and Jinggu white tea samples.

The heatmap analysis revealed the contents of 99 differential compounds in white tea from three different producing areas. In the heatmap, red signifies that compound levels were lower than the mean level across the three producing areas, while blue indicates that compound levels were higher than the mean level. These 99 differential compounds were categorized into three distinct groups, as illustrated in Fig. 3. The first category encompassed the majority of dimeric catechins (e.g., TF-3′-G, TF-DG, procyanidin C1, theasinensin F), all aroma precursors (e.g., benzyl glucoside, phenylethyl primeveroside, linalool primeveroside, geranyl primeveroside), most phenolic acids (e.g., lucuminic acid, 3,5-di-caffeoylquinic acid, strictinin, *trans*-cinnamic acid), most flavanols (e.g., EGCG, ECG, EGCG-4′'-O-Me, epiafzelechin), and most EPSFs (e.g., 8-C R-EGCG-cThea, 6-C R-EGCG-cThea, 8-C R-EGC-cThea, 6-C R-EC-cThea), all of which exhibited significantly higher contents in ZHWT samples compared to FDWT and JGWT samples. The second category included most amino acids (e.g., theanine, arginine, proline, aspartic acid) and three alkaloids (AMP, adenosine, and glycerophosphocholine) that were most abundant in FDWT samples, followed by ZHWT and JGWT samples. The third category was characterized by the presence of higher levels of most flavonol-O-glycosides (e.g., kaempferol-3-glucoside, quercetin-3-galactoside, quercetin-3-rutinoside, kaempferol-3-rutinoside) in JGWT samples, followed by ZHWT and FDWT samples. In an our previous study, we found that Yunnan white tea has higher contents of dimeric catechins (procyanidins, theasinensin, and theaflavin), organic acids, and lipids and lower contents of amino acids than FDWT (Gao et al., 2022, Gao et al., 2022), which was consistent with the above results. These findings indicate that the producing area significantly influences the compound levels in white tea samples, as compound contents in tea are closely associated with the geographical location and growth conditions of tea plants (Zhou et al., 2023). Furthermore, the influences caused by producing area were visually larger than that caused by storage duration from the heatmap because of more significant differences in color were observed among different producing areas than among different storage durations (Fig. 3). Therefore, we separately examined the impact of storage on ZHWT, FDWT, and JGWT.

### 3.3 Metabolite characteristics of white tea in different storage years

We constructed supervised PLS-DA score plots to investigate the impact of storage on white tea samples. Along principal component 1, the metabolites in white tea from three distinct producing areas exhibited a gradual and distinct pattern of change, as depicted in Fig. 1B-D. This pattern suggested that significant alterations in the compound profiles of white tea occur during storage. The reliability of the three PLS-DA models was confirmed by conducting permutation tests 100 times for each model. The results demonstrated that the intercepts for R^2^ and Q^2^ were 0.188 and −0.292 for the ZHWT PLS-DA model, 0.099 and −0.377 for the FDWT PLS-DA model, and 0.114 and −0.384 for the JGWT PLS-DA model. These results indicated the reliability of these three PLS-DA models, as illustrated in Fig. S1B-D. During storage, a total of 96, 66, and 59 compounds exhibited significant changes (VIP > 1 and *P*-value < 0.05) in ZHWT, FDWT, and JGWT, respectively, as shown in Fig. 2B.

#### 3.3.1 Common differential compounds of white tea in different storage years

Tea polyphenols, a significant class of active compounds in tea, primarily consist of catechins, flavonoids, and phenolic acids. These tea polyphenols offer numerous health benefits, such as anti-cancer, antibacterial, and antioxidant properties (Zhang, Qi, & Mine, 2019). The quantification of total tea polyphenols in white tea samples was conducted using the Folin-Ciocalteu colorimetric assay. As detailed in Table S2, the levels of tea polyphenols in white teas exhibited a decline after 16 years of storage in three distinct producing areas.

**Fig. 4:**
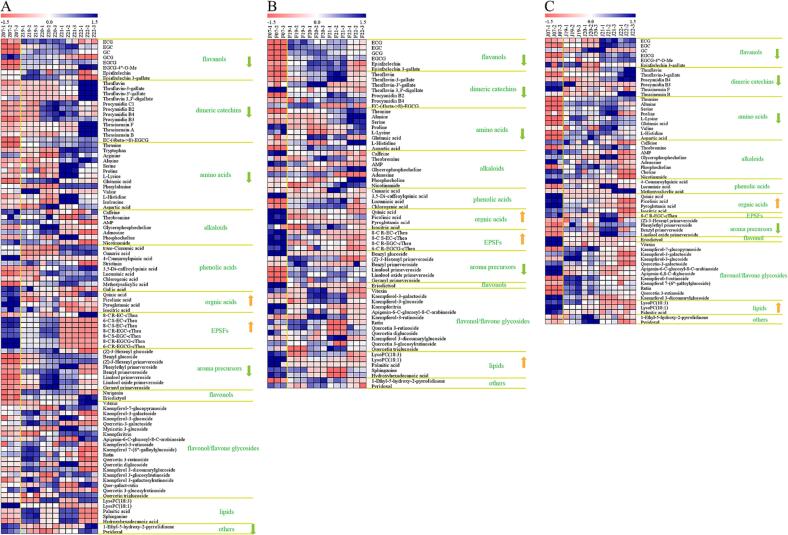
Heatmap analysis of the contents of storage duration-related differential compounds in (A) Zhenghe, (B) Fuding, and (C) Jinggu white tea samples.

Flavanols are a class of polyphenolic phytochemicals extensively present in tea leaves. They offer various health advantages and are primarily responsible for the characteristic puckering astringent taste (Chen et al., 2016, Cheng et al., 2020, Wu et al., 2022). These essential flavonoids in tea leaves can be transformed into dimeric catechins, such as TFs, procyanidins, and theasinensins. The majority of flavanols experienced a significant decrease with extended storage duration, as depicted in Figs. 4 and S3. Notably, the differentially abundant flavanols and dimeric catechins shared among ZHWT, FDWT, and JGWT during storage encompassed EGCG, ECG, EGC, epiafzelechin 3-gallate, TF, TF-3-G, and procyanidin B4. Among these compounds, EGC, ECG, and EGCG demonstrated reduced contents after 16 years of storage, aligning with our previous findings regarding the degradation of flavanols during prolonged storage (Dai et al., 2018, Xie et al., 2019). EGCG, for example, exhibited an 86.4 % decrease in ZHWT, 79.3 % in FDWT, and 74.4 % in JGWT samples after 16 years of storage. The overall content of flavanols decreased from 37.452 ± 1.067 to 21.758 ± 1.002 mg/g in ZHWT, from 56.694 ± 2.262 to 32.292 ± 2.378 mg/g in FDWT, and from 45.950 ± 2.740 to 34.053 ± 1.194 mg/g in JGWT following 16 years of storage, as shown in Table S2. These findings suggested that flavanols undergo degradation, polymerization, or interaction with other compounds during extended storage periods. In addition, flavanols are crucial astringent and bitter tastants in tea infusion (Chaturvedula & Prakash, 2011); that the significant decreases in the contents of flavanols is possibly the reason of the transformation of fresh, brisk, slightly astringent and bitter taste of 0-year white teas to the mellow taste of 16-year white teas (Table 1).

Previous research has indicated the close relationship between amino acids and the aroma compounds and refreshing taste of tea. The primary amino acid in tea is theanine, accompanied by glutamic acid and aspartic acid, which significantly contribute to the umami flavor. Sweetness enhancers include arginine and alanine. Interestingly, phenylalanine and tryptophan are associated with the bitter and astringent taste of tea (Liu et al., 2022, Tan et al., 2017, Zhu et al., 2020). In the present study, an overall decline in the total amino acid content was observed in white tea with extended storage duration. Notably, the significantly reduced amino acids included theanine, alanine, serine, lysine, glutamic acid, histidine, and aspartic acid. However, there was no consistent trend observed for proline during the storage period. Among these amino acids, the content of theanine exhibited the most substantial decrease, with ZHWT, FDWT, and JGWT samples decreasing to 91.7 %, 50.4 %, and 40.5 %, respectively, after prolonged storage. The quantitative determination of the total amino acid content in the white teas was conducted to explore the influence of storage on the teas. The reduction in amino acids post-storage may be attributed to oxidative degradation or their reactions to produce various aroma compounds (Chaturvedula & Prakash, 2011).

Flavonols and flavones in tea are predominantly found in the form of O-glycosides and C-glycosides based on linkage patterns (Zheng et al., 2018). Flavonol/flavone glycosides are present in minimal quantities in tea and are major contributors to the bitterness and astringency of tea (Ku, Kim, Park, Liu, & Lee, 2010). In this study, nine flavonol/flavone glycosides displayed irregular changes in content, including kaempferol-3-glucoside, kaempferol-3-galactoside, kaempferol-3-rutinoside, quercetin-3-rutinoside, kaempferol-3-dicoumarylglucoside, rutin, apigenin-6-C-glucosyl-8-C-arabinoside, vitexin, and eriodictyol (Figs. 4 and S4). These findings contrast with our earlier results, which revealed decreasing trends in most flavonol/flavone glycosides during white tea storage (Dai et al., 2018). This disparity may be attributed to variations in white tea types and experimental methods. The quantification of total flavonoids was conducted using a colorimetric method. Following 16 years of storage, the total flavonoid content in ZHWT, FDWT, and JGWT increased from 2.267 ± 0.104, 2.400 ± 0.028, and 3.483 ± 0.269 mg/g to 3.033 ± 0.134, 3.431 ± 0.294, and 4.213 ± 0.549 mg/g, respectively (Table S2).

It has been reported that tea contains a substantial amount of alkaloids, particularly purine alkaloids. Among them, caffeine, with the highest content, is responsible for the bitterness and astringency of tea infusion. Caffeine plays a significant role in enhancing the flavor of tea compared to catechins, making a substantial contribution to tea quality (Chen et al., 2010, Yang et al., 2007, Ye et al., 2018). The levels of caffeine, AMP, and adenosine increased, whereas theobromine, glycerophosphocholine, phosphocholine, and nicotinamide showed no significant changes during extended storage periods (Figs. 4 and S4). As indicated in Table S2, the quantification of caffeine content was performed using a colorimetric method.

Aroma precursor compounds, primarily aroma primeverosides, can release aroma compounds during tea production and brewing (Ho, Zheng, & Li, 2015). In this study, the levels of (Z)-3-hexenyl primeveroside, benzyl primeveroside, and linalool oxide primeveroside were highest in the long-term storage white teas and lowest in the 1-year white teas, which indicated that (Z)-3-hexenol, benzyl alcohol, and linalool oxide may involve in the aroma formation of long-term stored white tea. Furthermore, these three aroma primeverosides were notably more abundant in ZHWT samples than in FDWT and JGWT samples.

The degree of water extract and water-soluble sugars has an impact on the tea brewing concentration and taste characteristics of tea infusion, serving as crucial indicators to assess tea quality. The level of water extract exhibited a slight reduction in samples of white teas from the three different producing areas after 16 years of storage, while water-soluble sugars did not undergo significant changes in this study (Table S2).

#### 3.3.2 EPSFs of white tea in different storage years

**Fig. 5:**
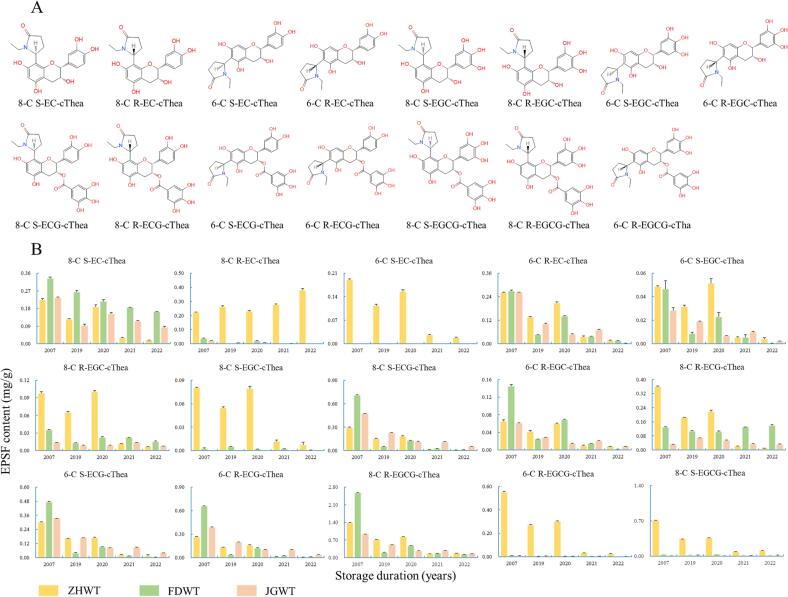
(A) The chemical structure of 15 EPSFs in white teas; (B) The changing patterns of 15 EPSF compounds in Zhenghe, Fuding, and Jinggu white tea samples during storage.

*N*-ethyl-2-pyrrolidinone-substituted flavanols (EPSFs), as typical representatives of flavoalkaloids, are compounds with a characteristic flavonoid structure and a 5-membered ring substituting the A-ring in their configuration (Cheng et al., 2018). The formation of EPSF compounds involves a sequence of reactions: initially, theanine undergoes Strecker degradation and spontaneous cyclization reactions, converting it into 1-ethyl-5-hydroxy-2-pyrrolidinone. This compound then reacts with the C-6 and/or C-8 positions of flavanols to produce EPSFs. Due to the chiral nature of the carbon atom in the flavanol that links with the pyrrole ring, EPSF compounds exist in two configurations, S- and R-configuration (Meng et al., 2018). In addition, EPSFs have garnered significant attention for their potential health benefits. For instance, EPSF compounds have demonstrated a protective effect on human microvascular endothelial cells and exhibit anti-inflammatory properties by regulating the NF-κB signaling pathway, and other strong biological activities (Shi et al., 2021, Xie et al., 2019). In our previous studies, EPSFs showed linear correlations with storage duration of white, green, black, and oolong teas (Chen et al., 2021, Dai et al., 2020, Dai et al., 2018, Peng et al., 2021). In this study, we identified fifteen EPSF compounds (8-C R-EC-cThea, 6-C S-EC-cThea, 6-C R-EC-cThea, 8-C S-EC-cThea, 6-C S-EGC-cThea, 8-C R-EGC-cThea, 8-C S-EGC-cThea, 6-C R-EGC-cThea, 8-C S-ECG-cThea, 8-C R-ECG-cThea, 6-C S-ECG-cThea, 6-C R-ECG-cThea, 8-C R-EGCG-cThea, 6-C R-EGCG-cThea, and 8-C S-EGCG-cThea) in white teas (Fig. 5A and Fig. S1-B), and the contents of nearly all EPSF compounds displayed an increasing trend after extended storage (Fig. 5B). After 16 years of storage, the total content of EPSFs in ZHWT, FDWT, and JGWT increased from 0.807 ± 0.038, 0.504 ± 0.010, and 0.426 ± 0.023 mg/g to 5.050 ± 0.092, 5.438 ± 0.050, and 2.783 ± 0.026 mg/g, respectively. The ratio of EPSFs formed by ECG/EGCG to EC/EGC was 3.24, 5.27, and 3.48 in ZHWT, FDWT, and JGWT samples after 16 years of storage, respectively. These findings suggested that galloylated flavanols (ECG and EGCG) had a greater tendency to form EPSFs with theanine than non-galloylated flavanols (EC and EGC) during storage because of the higher contents of galloylated flavanols than of non-galloylated flavanols in white tea. The 8-C R-EGCG-cThea/8-C S-EGCG-cThea content ratio was 1.96, 92.49, and 177.13 in ZHWT, FDWT, and JGWT samples after 16 years of storage, respectively. This indicated that theanine and EGCG were more likely to generate R-configuration EPSFs during storage in ZHWT, FDWT and JGWT samples. In addition, the 8-C EPSF compounds showed higher content than that of 6-C EPSF compounds, which indicated that theanine is inclined to attack and react with the 8-C atom of EGCG molecule during storage.

## 4 Conclusion

In summary, a comprehensive examination of non-volatile compounds in white teas originating from different regions and storage durations was carried out using nontargeted metabolomics and quantitative analysis. Across these assessments, a total of 83 (ZHWT vs. FDWT), 89 (ZHWT vs. JGWT) and 75 (FDWT vs. JGWT) differential compounds were identified from three different producing areas. Among these compounds, amino acids and flavonol/flavone glycosides exhibited the highest number of variations. When assessing white teas of differing storage durations, it was observed that the levels of flavanols, dimeric catechins, and amino acids declined with extended storage, while all EPSF compounds, caffeine, AMP, and adenosine increased. Galloylated flavanols (ECG and EGCG) exhibited a greater tendency to form EPSFs with theanine compared to nongalloylated flavanols (EC and EGC) during storage. Furthermore, theanine and EGCG were more inclined to generate S-configuration EPSF during storage in FDWT and JGWT samples, while R-configuration EPSF was more readily formed in ZHWT samples. The contents of flavonol/flavone glycosides and theobromine displayed irregular patterns of change. These findings offer a comprehensive insight into the alterations in compound composition in white tea during storage, which can be valuable for the proper storage of white tea.

## CRediT authorship contribution statement

**Zewen Chen:** Writing – original draft, Methodology, Formal analysis, Data curation. **Weidong Dai:** Writing – review & editing, Resources, Methodology, Investigation, Formal analysis, Conceptualization. **Mengfan Xiong:** Methodology, Investigation. **Jianjian Gao:** Methodology, Investigation. **Hongjie Zhou:** Writing – review & editing, Project administration, Conceptualization. **Dan Chen:** Writing – original draft, Methodology, Investigation, Formal analysis. **Yali Li:** Writing – review & editing, Methodology, Investigation.

## Declaration of competing interest

The authors declare that they have no known competing financial interests or personal relationships that could have appeared to influence the work reported in this paper.

## Data Availability

Data will be made available on request.
