# Supplementary material for: Metabolomics investigation of the chemical variations in white teas with different producing areas and storage durations
Source: Food Chem X. 2024 Jan 6;21:101127. doi: 10.1016/j.fochx.2024.101127 (PMC10825419; doi:10.1016/j.fochx.2024.101127)
Supplement: Supplementary data 1 [file mmc1.docx]

**Metabolomics investigation of the chemical variations in white teas with different producing areas and storage durations**

Zewen Chen^1,#^, Weidong Dai^2,#^, Mengfan Xiong^1^, Jianjian Gao^2^, Hongjie Zhou^1,^*, Dan Chen^2,^**, Yali Li^1,^***

^1^ College of tea, Yunnan Agricultural University, Kunming, Yunnan, 650201, China

^2^ Tea Research Institute, Chinese Academy of Agricultural Sciences, Hangzhou, Zhejiang 310008, China

* Corresponding Author: Tel.: +86 13708478322. E-mail: 1051195348@qq.com

** Corresponding author: Tel.: +86 571 87967281. E-mail: chendan@tricaas.com.

***Corresponding author: Tel.: +86 13759169886. E-mail: 595778901@qq.com.

^#^ These authors contributed equally to this work.

Table S1. Summary of putatively identified non-volatile compounds

| NO. | RT [min] | | | *m/z* |  | Compound | Fragments of MS2 | Referred database | VIP | P-value |
| --- | --- | --- | --- | --- | --- | --- | --- | --- | --- | --- |
|  | |  | **Flavanols** | | | | | | | |
| 1 | 9.988 | | | 291.0860 |  | EC* | - | - | 0.99 | 0.00 |
| 2 | 7.866 | | | 291.0861 |  | C* | - | - | 0.77 | 0.03 |
| 3 | 13.101 | | | 443.0964 |  | ECG* | - | - | 1.03 | 0.00 |
| 4 | 7.406 | | | 307.0808 |  | EGC* | - | - | 1.24 | 0.00 |
| 5 | 4.839 | | | 307.0809 |  | GC* | - | - | 1.62 | 0.00 |
| 6 | 12.018 | | | 459.0915 |  | GCG* | - | - | 1.45 | 0.00 |
| 7 | 10.218 | | | 459.0915 |  | EGCG* | - | - | 1.31 | 0.00 |
| 8 | 12.500 | | | 473.1071 |  | EGCG-4''-O-Me* | - | - | 1.77 | 0.00 |
| 9 | 11.786 | | | 275.0910 |  | Epiafzelechin* | - | - | 1.39 | 0.00 |
| 10 | 12.954 | | | 611.1021 |  | EGC 3,5-digallate | 457, 169, 333 | HMDB | 0.68 | 0.00 |
| 11 | 14.489 | | | 427.1016 |  | Epiafzelechin 3-gallate* | - | - | 1.57 | 0.00 |
|  | |  | **Dimeric catechins** | | | | | | | |
| 12 | 16.417 | | | 565.1333 |  | Theaflavin* | - | - | 1.35 | 0.00 |
| 13 | 16.790 | | | 717.1437 |  | Theaflavin-3-gallate* | - | - | 1.14 | 0.00 |
| 14 | 17.027 | | | 717.1436 |  | Theaflavin-3'-gallate* | - | - | 1.32 | 0.00 |
| 15 | 17.002 | | | 869.1547 |  | Theaflavin 3,3'-digallate* | - | - | 1.51 | 0.00 |
| 16 | 9.675 | | | 867.2118 |  | Procyanidin C1* | - | - | 1.78 | 0.00 |
| 17 | 8.838 | | | 579.1487 |  | Procyanidin B2* | - | - | 1.04 | 0.00 |
| 18 | 7.561 | | | 579.1487 |  | Procyanidin B4* | - | - | 1.24 | 0.00 |
| 19 | 9.264 | | | 579.1489 |  | Procyanidin B3* | - | - | 1.82 | 0.00 |
| 20 | 11.470 | | | 899.1655 |  | Theasinensin F* | - | - | 1.52 | 0.00 |
| 21 | 9.707 | | | 915.1601 |  | Theasinensin A* | - | - | 1.74 | 0.00 |
| 22 | 6.539 | | | 763.1493 |  | Theasinensin B* | - | - | 1.37 | 0.00 |
| 23 | 11.102 | | | 731.1592 |  | EC-(4alpha->8)-ECG | 441, 273, 153 | HMDB | 0.89 | 0.00 |
| 24 | 9.982 | | | 747.1541 |  | EC-(4beta->8)-EGCG | 621, 153, 457 | HMDB | 1.57 | 0.00 |
|  | |  | **Amino acids** | | | | | | | |
| 25 | 1.261 | | | 175.1077 |  | Theanine* | - | - | 1.59 | 0.00 |
| 26 | 5.476 | | | 205.0971 |  | Tryptophan* | - | - | 1.60 | 0.00 |
| 27 | 0.806 | | | 213.0746 |  | Arginine* | - | - | 1.60 | 0.00 |
| 28 | 0.845 | | | 90.0553 |  | Alanine* | - | - | 1.46 | 0.00 |
| 29 | 0.839 | | | 106.0499 |  | Serine* | - | - | 1.08 | 0.00 |
| 30 | 0.903 | | | 116.0708 |  | Proline* | - | - | 1.39 | 0.00 |
| 31 | 0.844 | | | 147.0764 |  | Glutamine* | - | - | 0.79 | 0.00 |
| 32 | 0.767 | | | 147.1128 |  | L-Lysine* | - | - | 1.50 | 0.00 |
| 33 | 0.858 | | | 148.0603 |  | Glutamic acid* | - | - | 1.19 | 0.00 |
| 34 | 3.245 | | | 166.0862 |  | Phenylalanine* | - | - | 1.44 | 0.00 |
| 35 | 0.878 | | | 118.0864 |  | Valine* | - | - | 1.41 | 0.00 |
| 36 | 0.760 | | | 156.0767 |  | L-Histidine* | - | - | 1.06 | 0.00 |
| 37 | 1.959 | | | 132.1020 |  | Isoleucine* | - | - | 1.80 | 0.00 |
| 38 | 1.773 | | | 132.1020 |  | Leucine* | - | - | 0.99 | 0.00 |
| 39 | 0.838 | | | 133.0608 |  | Asparaginate* | - | - | 0.79 | 0.00 |
| 40 | 0.846 | | | 134.0447 |  | Aspartic acid* | - | - | 1.62 | 0.00 |
| 41 | 0.851 | | | 120.0656 |  | L-Threonine* | - | - | 0.96 | 0.00 |
|  | |  | **Alkaloids** | | | | | | | |
| 42 | 8.319 | | | 195.0876 |  | Caffeine* | - | - | 1.24 | 0.00 |
| 43 | 4.972 | | | 181.0720 |  | Theobromine* | - | - | 1.43 | 0.00 |
| 44 | 1.257 | | | 348.0696 |  | AMP* | - | - | 1.02 | 0.00 |
| 45 | 1.492 | | | 166.0724 |  | 7-Methylguanine | 165, 148 | HMDB | 0.67 | 0.05 |
| 46 | 0.855 | | | 258.1098 |  | Glycerophosphocholine | 78, 182, 152 | HMDB | 1.01 | 0.00 |
| 47 | 1.990 | | | 268.1037 |  | Adenosine* | - | - | 1.07 | 0.00 |
| 48 | 6.051 | | | 298.0965 |  | 5'-Methylthioadenosine | 134, 135 | HMDB | 0.76 | 0.00 |
| 49 | 1.988 | | | 314.0914 |  | (S)-5'-Deoxy-5'-(methylsulfinyl)adenosine | 134, 250 | HMDB | 0.95 | 0.00 |
| 50 | 0.846 | | | 184.0733 |  | Phosphocholine | 86, 124 | HMDB | 1.35 | 0.00 |
| 51 | 0.832 | | | 104.1072 |  | Choline* | - | - | 1.13 | 0.00 |
| 52 | 0.893 | | | 134.0813 |  | N-Lactoyl ethanolamine | 45, 134, 116 | HMDB | 0.70 | 0.04 |
| 53 | 0.923 | | | 136.0618 |  | Adenine | 107, 117, 92 | HMDB | 0.82 | 0.00 |
| 54 | 0.884 | | | 127.0503 |  | Thymine | 41, 125 | HMDB | 0.72 | 0.00 |
| 55 | 1.340 | | | 123.0554 |  | Nicotinamide | 123, 80 | HMDB | 1.18 | 0.00 |
|  | |  | **Phenolic acids** | | | | | | | |
| 56 | 3.553 | | | 149.0596 |  | trans-Cinnamic acid | 77, 103 | HMDB | 1.68 | 0.00 |
| 57 | 1.700 | | | 165.0546 |  | Cumaric acid* | - | - | 1.77 | 0.00 |
| 58 | 7.428 | | | 339.1069 |  | 4-Coumaroylquinic acid | 147, 119, 101 | HMDB | 1.01 | 0.00 |
| 59 | 10.122 | | | 339.1070 |  | 3-Coumaroylquinic acid | 147, 119, 215 | HMDB | 0.96 | 0.00 |
| 60 | 11.721 | | | 467.0813 |  | Digalloylglucose | 153, 171, 135 | HMDB | 1.45 | 0.00 |
| 61 | 9.468 | | | 475.0865 |  | Chicoric acid | 161, 249, 163 | HMDB | 0.68 | 0.01 |
| 62 | 8.602 | | | 635.0866 |  | Strictinin | 153, 377, 127 | HMDB | 1.33 | 0.00 |
| 63 | 14.365 | | | 517.1335 |  | 3,5-Di-caffeoylquinic acid | 353, 173, 179 | HMDB | 1.21 | 0.00 |
| 64 | 10.814 | | | 469.1310 |  | Lucuminic acid | 313, 295, 59 | HMDB | 1.46 | 0.00 |
| 65 | 3.147 | | | 345.0810 |  | Theogallin* | - | - | 0.33 | 0.63 |
| 66 | 8.130 | | | 355.1018 |  | Chlorogenic acid* | - | - | 1.24 | 0.00 |
| 67 | 7.402 | | | 169.0495 |  | Methoxysalicylic acid | 108, 152, 167 | HMDB | 1.51 | 0.00 |
| 68 | 2.465 | | | 171.0287 |  | Gallic acid* | - | - | 1.31 | 0.00 |
|  | |  | **Orgnic acids** | | | | | | | |
| 69 | 0.910 | | | 193.0706 |  | Quinic acid* | - | - | 1.06 | 0.01 |
| 70 | 1.268 | | | 124.0394 |  | Picolinic acid | 117, 64, 45 | HMDB | 1.12 | 0.00 |
| 71 | 0.927 | | | 130.0863 |  | Pipecolic acid* | - | - | 0.23 | 0.63 |
| 72 | 1.258 | | | 130.0499 |  | Pyroglutamic acid* | - | - | 1.07 | 0.00 |
| 73 | 0.836 | | | 169.0583 |  | Vanillic acid | 108, 152 | HMDB | 0.70 | 0.01 |
| 74 | 1.367 | | | 174.0762 |  | 2,6-Piperidinedicarboxylic acid | 122, 108 | HMDB | 0.94 | 0.00 |
| 75 | 13.150 | | | 197.1170 |  | 2,2'-(3-methylcyclohexane-1,1-diyl)diacetic acid | 113, 85 | HMDB | 0.73 | 0.08 |
| 76 | 1.355 | | | 215.0162 |  | Isocitric acid | 111, 173, 85 | HMDB | 1.46 | 0.00 |
| 77 | 4.505 | | | 220.1179 |  | Pantothenic acid | 88, 146, 218 | HMDB | 0.28 | 0.68 |
|  | |  | **EPSFs** | | | | | | | |
| 78 | 13.633 | | | 402.1549 |  | 8-C R-EC-cThea | 250, 205, 262, 384 | Database | 1.74 | 0.00 |
| 79 | 12.770 | | | 402.1558 |  | 6-C S-EC-cThea | 250, 205, 262, 384 | Database | 1.36 | 0.00 |
| 80 | 13.120 | | | 402.1572 |  | 6-C R-EC-cThea | 250, 205, 262, 384 | Database | 0.27 | 0.57 |
| 81 | 13.350 | | | 402.1573 |  | 8-C S-EC-cThea | 250, 205, 262, 384 | Database | 1.87 | 0.00 |
| 82 | 11.310 | | | 418.1479 |  | 6-C S-EGC-cThea | 250, 205, 400, 262 | Database | 0.39 | 0.29 |
| 83 | 11.660 | | | 418.1489 |  | 8-C R-EGC-cThea | 250, 205, 400, 262 | Database | 1.02 | 0.00 |
| 84 | 10.690 | | | 418.1489 |  | 8-C S-EGC-cThea | 250, 205, 400, 262 | Database | 1.36 | 0.00 |
| 85 | 11.410 | | | 418.1491 |  | 6-C R-EGC-cThea | 250, 205, 400, 262 | Database | 0.55 | 0.19 |
| 86 | 14.600 | | | 554.1645 |  | 8-C S-ECG-cThea | 250, 262, 384, 205 | Database | 0.25 | 0.63 |
| 87 | 14.740 | | | 554.1648 |  | 8-C R-ECG-cThea | 250, 262, 384, 205 | Database | 0.71 | 0.00 |
| 88 | 14.330 | | | 554.1649 |  | 6-C S-ECG-cThea | 250, 262, 384, 205 | Database | 0.11 | 0.94 |
| 89 | 14.470 | | | 554.1652 |  | 6-C R-ECG-cThea | 250, 262, 384, 205 | Database | 0.27 | 0.65 |
| 90 | 13.370 | | | 570.1595 |  | 8-C R-EGCG-cThea* | - | - | 1.35 | 0.00 |
| 91 | 12.810 | | | 570.1597 |  | 6-C R-EGCG-cThea | 250, 262, 400, 205 | Database | 1.27 | 0.00 |
| 92 | 13.210 | | | 570.1598 |  | 8-C S-EGCG-cThea* | - | - | 0.62 | 0.20 |
|  | |  | **Aroma precursors** | | | | | | | |
| 93 | 14.106 | | | 285.1305 |  | (Z)-3-Hexenyl glucoside* | - | - | 1.74 | 0.00 |
| 94 | 8.356 | | | 293.0991 |  | Benzyl glucoside* | - | - | 1.76 | 0.00 |
| 95 | 12.053 | | | 417.1724 |  | (Z)-3-Hexenyl primeveroside* | - | - | 1.71 | 0.00 |
| 96 | 11.632 | | | 439.1568 |  | Phenylethyl primeveroside* | - | - | 1.81 | 0.00 |
| 97 | 9.604 | | | 425.1411 |  | Benzyl primeveroside* | - | - | 1.32 | 0.00 |
| 98 | 17.144 | | | 471.2193 |  | Linalool primeveroside* | - | - | 1.74 | 0.00 |
| 99 | 14.062 | | | 487.2143 |  | Linalool oxide primeveroside | 313, 331, 57 | HMDB | 1.63 | 0.00 |
| 100 | 16.891 | | | 471.2195 |  | Geranyl primeveroside* | - | - | 1.71 | 0.00 |
|  | |  | **Flavonols** | | | | | | | |
| 101 | 13.102 | | | 273.0753 |  | Narigenin | 119, 151, 107 | HMDB | 1.81 | 0.00 |
| 102 | 18.295 | | | 287.0547 |  | Kaempferol | 117, 227, 158 | HMDB | 0.82 | 0.00 |
| 103 | 12.501 | | | 289.0701 |  | Eriodictyol | 255, 229, 135 | HMDB | 1.64 | 0.00 |
| 104 | 16.709 | | | 303.0494 |  | Quercetin | 271, 151, 121 | HMDB | 0.47 | 0.08 |
| 105 | 12.045 | | | 319.0444 |  | Myricetin | 317, 138 | HMDB | 0.76 | 0.08 |
|  | |  | **Flavonol/flavone glycosides** | | | | | | | |
| 106 | 13.378 | | | 433.1122 |  | Isovitexin* | - | - | 0.90 | 0.00 |
| 107 | 13.171 | | | 433.1124 |  | Vitexin* | - | - | 1.38 | 0.00 |
| 108 | 13.724 | | | 449.1070 |  | Kaempferol-7-glucopyranoside | 285, 184 | HMDB | 1.22 | 0.00 |
| 109 | 14.116 | | | 449.1072 |  | Kaempferol-3-galactoside* | - | - | 1.30 | 0.00 |
| 110 | 14.442 | | | 449.1072 |  | Kaempferol-3-glucoside* | - | - | 1.26 | 0.00 |
| 111 | 13.364 | | | 465.1022 |  | Quercetin-3-galactoside* | - | - | 1.23 | 0.00 |
| 112 | 13.552 | | | 465.1022 |  | Quercetin-3-glucoside* | - | - | 0.51 | 0.32 |
| 113 | 12.041 | | | 481.0972 |  | Myricetin-3-galactoside* | - | - | 0.34 | 0.26 |
| 114 | 12.235 | | | 481.0972 |  | Myricetin-3-glucoside* | - | - | 1.58 | 0.00 |
| 115 | 13.333 | | | 579.1700 |  | Kaempferitrin | 287, 433 | HMDB | 1.69 | 0.00 |
| 116 | 16.925 | | | 595.1436 |  | Kaempferol-3-coumaroyglucoside | 153, 139 | HMDB | 0.97 | 0.00 |
| 117 | 17.463 | | | 595.1436 |  | 2''-O-trans-p-Coumaroylastragalin | 284, 255 | HMDB | 0.30 | 0.62 |
| 118 | 12.201 | | | 565.1543 |  | Apigenin-6-C-glucosyl-8-C-arabinoside | 447, 281 | HMDB | 1.57 | 0.00 |
| 119 | 10.840 | | | 595.1647 |  | Apigenin-6,8-C-diglucoside* | - | - | 1.08 | 0.00 |
| 120 | 14.165 | | | 595.1648 |  | Kaempferol-3-rutinoside* | - | - | 1.21 | 0.00 |
| 121 | 13.612 | | | 601.1178 |  | Kaempferol-7-(6''-galloylglucoside) | 467, 453 | HMDB | 1.44 | 0.00 |
| 122 | 14.212 | | | 601.1179 |  | Kaempferol-3-(6''-galloylglucoside) | 451, 424 | HMDB | 0.96 | 0.00 |
| 123 | 13.138 | | | 611.1592 |  | Rutin* | - | - | 1.62 | 0.00 |
| 124 | 13.291 | | | 611.1592 |  | Quercetin-3-rutinoside* | - | - | 1.42 | 0.00 |
| 125 | 12.079 | | | 627.1545 |  | Quercetin diglucoside | 463, 462 | HMDB | 1.79 | 0.00 |
| 126 | 19.730 | | | 741.1800 |  | Kaempferol-3-dicoumarylglucoside | 621, 153 | HMDB | 1.10 | 0.00 |
| 127 | 13.726 | | | 757.2170 |  | Kaempferol-3-glucosylrutinoside | 287, 488 | HMDB | 1.49 | 0.00 |
| 128 | 13.312 | | | 757.2171 |  | Kaempferol-3-galactosylrutinoside | 593, 592 | HMDB | 1.41 | 0.00 |
| 129 | 12.603 | | | 773.2120 |  | Quer-galact-rutin | 303, 465 | HMDB | 1.81 | 0.00 |
| 130 | 12.892 | | | 773.2120 |  | Quercetin-3-glucosylrutinoside | 300, 271 | HMDB | 1.22 | 0.00 |
| 131 | 11.634 | | | 789.2070 |  | Quercetin triglucoside | 300, 255 | HMDB | 1.37 | 0.00 |
|  | |  | **Lipids** | | | | | | | |
| 132 | 21.991 | | | 359.3150 |  | MG(18:0) | 99, 81 | HMDB | 0.61 | 0.20 |
| 133 | 21.016 | | | 353.2682 |  | MG(18:3) | 73, 59 | HMDB | 0.76 | 0.00 |
| 134 | 21.614 | | | 496.3392 |  | LysoPC(16:0) | 104, 146 | HMDB | 0.93 | 0.00 |
| 135 | 21.102 | | | 518.3235 |  | LysoPC(18:3) | 357, 104 | HMDB | 1.14 | 0.00 |
| 136 | 21.379 | | | 520.3391 |  | LysoPC(18:2) | 337, 104 | HMDB | 0.90 | 0.00 |
| 137 | 21.778 | | | 522.3546 |  | LysoPC(18:1) | 184, 104 | HMDB | 1.21 | 0.00 |
| 138 | 20.098 | | | 274.2738 |  | Palmitic acid | 255, 355 | HMDB | 1.30 | 0.00 |
| 139 | 20.603 | | | 318.2998 |  | Phytosphingosine | 264, 121, 135 | HMDB | 0.97 | 0.00 |
| 140 | 21.308 | | | 277.2159 |  | Stearidonic acid | 79, 93, 67 | HMDB | 0.71 | 0.00 |
| 141 | 0.920 | | | 204.1228 |  | Acetylcarnitine | 85, 144, 60 | HMDB | 0.98 | 0.00 |
| 142 | 17.783 | | | 251.1251 |  | Ubiquinone-1 | 195, 211, 193 | HMDB | 0.70 | 0.00 |
| 143 | 22.419 | | | 607.2535 |  | Phaeophorbide b | 531, 501 | HMDB | 0.65 | 0.01 |
| 144 | 20.577 | | | 302.3050 |  | Sphinganine | 284, 254, 60 | HMDB | 1.14 | 0.00 |
| 145 | 20.198 | | | 290.2687 |  | Hydroxyhexadecanoic acid | 255, 237 | HMDB | 1.11 | 0.00 |
| 146 | 20.988 | | | 256.2633 |  | Palmitic amide | 57, 55, 43 | HMDB | 1.00 | 0.00 |
| 147 | 21.582 | | | 535.2693 |  | Pyrophaeophorbide a | 489, 515, 473 | HMDB | 0.40 | 0.21 |
|  | |  | **Others** | | | | | | | |
| 148 | 2.823 | | | 130.0863 |  | 1-Ethyl-5-hydroxy-2-pyrrolidinone* | - | - | 1.55 | 0.00 |
| 149 | 0.885 | | | 203.0527 |  | Glucose | 100, 139 | HMDB | 0.58 | 0.02 |
| 150 | 19.336 | | | 181.1223 |  | Dihydroactinidiolide | 151, 135 | HMDB | 0.49 | 0.36 |
| 151 | 1.417 | | | 168.0632 |  | Pyridoxal | 138, 107 | HMDB | 1.22 | 0.00 |
| 152 | 9.977 | | | 411.1618 |  | Hydroxyjasmonic acid glucoside | 353, 371, 311 | HMDB | 0.95 | 0.00 |
| 153 | 1.337 | | | 337.1600 |  | Theanine glucoside | 288, 145 | HMDB | 0.63 | 0.01 |
| 154 | 9.245 | | | 257.1380 |  | 2-[4-(3-Hydroxypropyl)-2-methoxyphenoxy]-1,3-propanediol | 181, 165, 149 | HMDB | 0.91 | 0.00 |

Note: * Meaning the compounds were identified by authentic compounds, others were deduced by mass or database.

Table S2. Contents of main compounds in Zhenghe, Fuding, Jinggu white tea samples during storage.

| Samples | Caffeine (%) | Total flavonoids (mg/g) | Tea Polyphenols (%) | Flavanols (mg/g) | Amino acids (%) | Water-soluble sugars (%) | Water extracts (%) |
| --- | --- | --- | --- | --- | --- | --- | --- |
| Z07 | 3.269±0.136a | 3.033±0.134a | 15.097±0.568b,c | 21.758±1.002d | 0.048±0.002a | 0.011±0.003a | 0.385±0.012d |
| Z19 | 3.241±0.544a | 2.364±0.135c | 16.099±0.670b | 29.075±5.316b,c | 0.048±0.002a | 0.015±0.001a | 0.410±0.002c |
| Z20 | 3.097±0.091a | 2.467±0.025c | 14.096±0.348c,d | 26.647±3.523c | 0.050±0.001a | 0.018±0.005a | 0.429±0.007b |
| Z21 | 3.096±0.098a | 2.395±0.098b | 13.079±0.984d | 33.992±1.483a,b | 0.048±0.013b | 0.013±0.001a | 0.455±0.007a |
| Z22 | 3.238±0.165a | 2.267±0.104b | 17.519±0.952a | 37.452±1.067a | 0.049±0.001a | 0.014±0.002a | 0.432±0.011b |
| F07 | 3.135±0.113a | 3.431±0.294a | 14.081±1.169b | 32.292±2.378d | 0.052±0.001c | 0.004±0.000d | 0.381±0.003c |
| F19 | 3.192±0.368a | 2.651±0.328d | 14.753±0.927b | 64.706±5.889a | 0.058±0.001b | 0.090±0.000c | 0.403±0.003b |
| F20 | 2.697±0.032b | 2.796±0.077b | 15.112±1.637b | 56.572±3.598b | 0.052±0.002c | 0.012±0.002b | 0.440±0.012a |
| F21 | 2.850±0.022a,b | 2.629±0.103c | 17.339±0.417a | 48.742±4.630c | 0.057±0.003b | 0.014±0.002a,b | 0.433±0.015a |
| F22 | 2.994±0.073a,b | 2.400±0.028d | 15.217±0.247b | 56.694±2.262b | 0.067±0.002a | 0.015±0.001a | 0.426±0.015a |
| J07 | 2.821±0.079b,c | 4.213±0.549a | 13.812±0.679d | 34.053±1.194b | 0.034±0.000c | 0.007±0.001b | 0.393±0.010c |
| J19 | 3.008±0.070a | 3.365±0.414b | 16.472±0.679b,c | 35.874±3.696b | 0.046±0.002b | 0.002±0.001c | 0.428±0.007b |
| J20 | 2.907±0.076a,b | 3.403±0.091b | 15.037±1.020c,d | 34.903±1.549b | 0.049±0.001a | 0.003±0.001c | 0.467±0.005a |
| J21 | 3.015±0.064a | 3.952±0.167a,b | 17.265±0.822b | 46.132±5.941a | 0.048±0.001a | 0.010±0.003a | 0.446±0.020a,b |
| J22 | 2.714±0.013c | 3.483±0.269b | 19.522±1.541a | 45.950±2.740a | 0.044±0.001b | 0.011±0.001a | 0.428±0.012b |

Note: (Z: Zhenghe white tea samples; F: Fuding white tea samples; Y: Jinggu white tea samples).


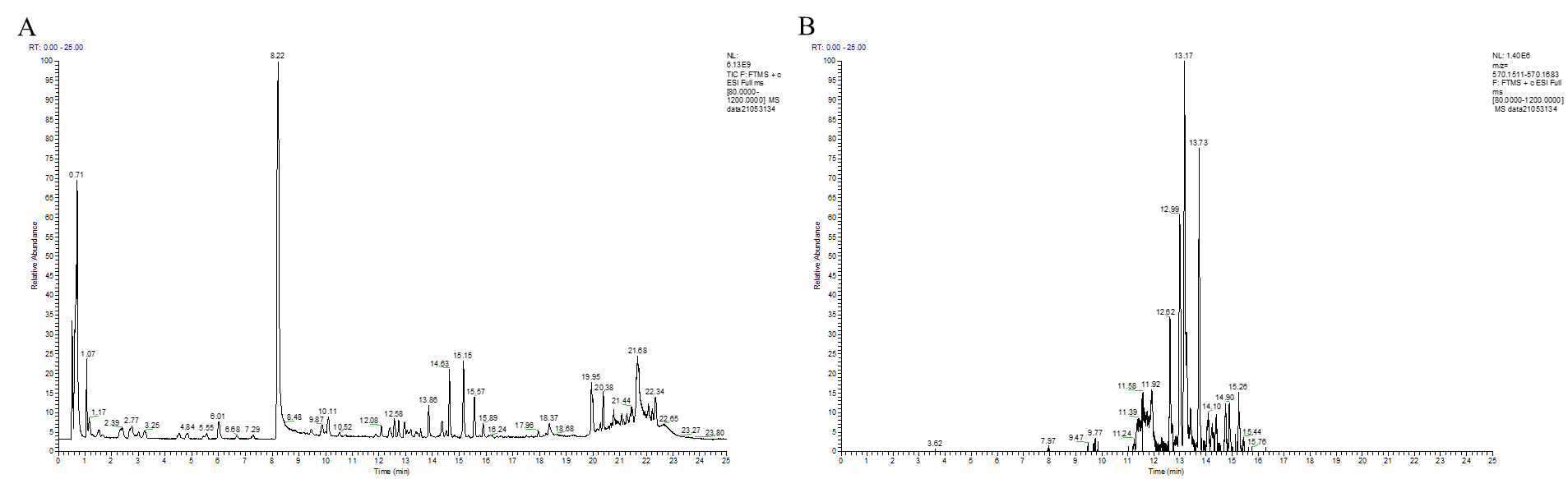


Figure S1. (A) Typical total ion current (TIC) chromatogram of ZHWT samples analyzed in the ESI^+^ mode; (B) typical extracted ion chromatogram (EIC) of EPSF compound (EGCG-cThea is shown as an example) in ZHWT samples.


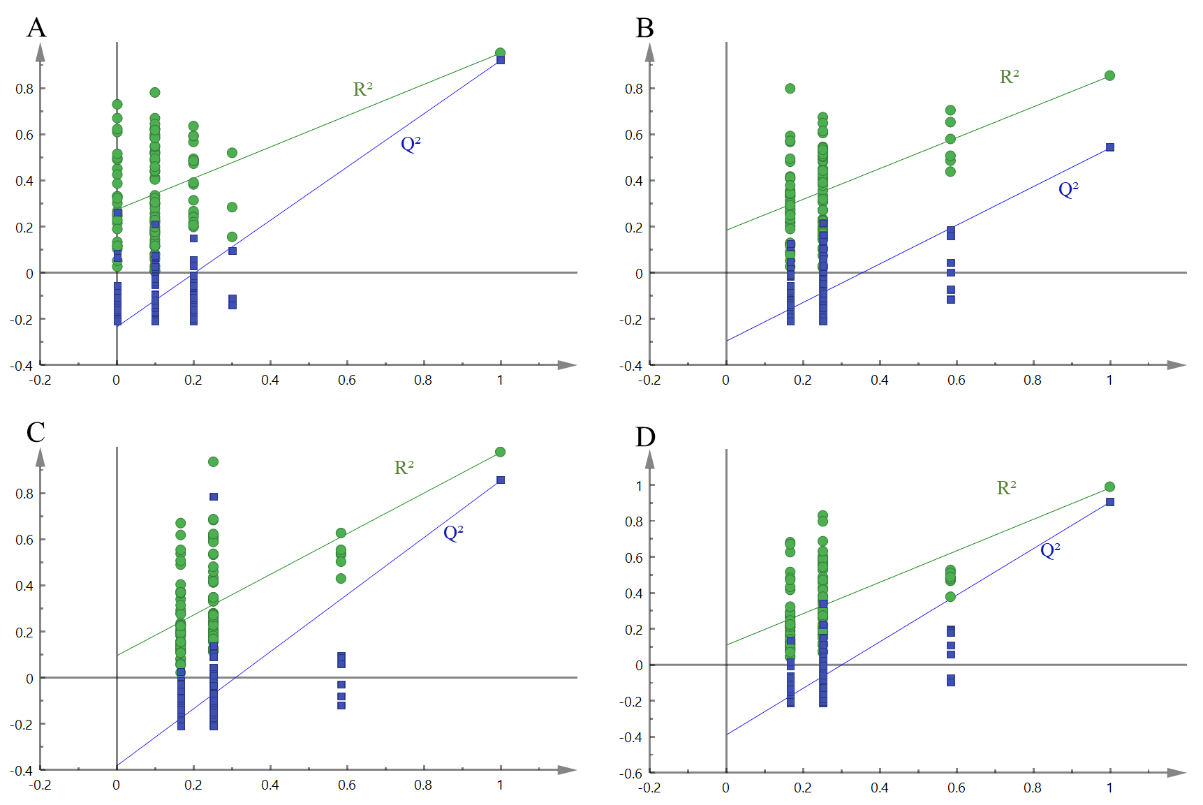


Figure S2. Correspond to the cross-verification plots of 100 times permutation tests in four PLS-DA modes, respectively.


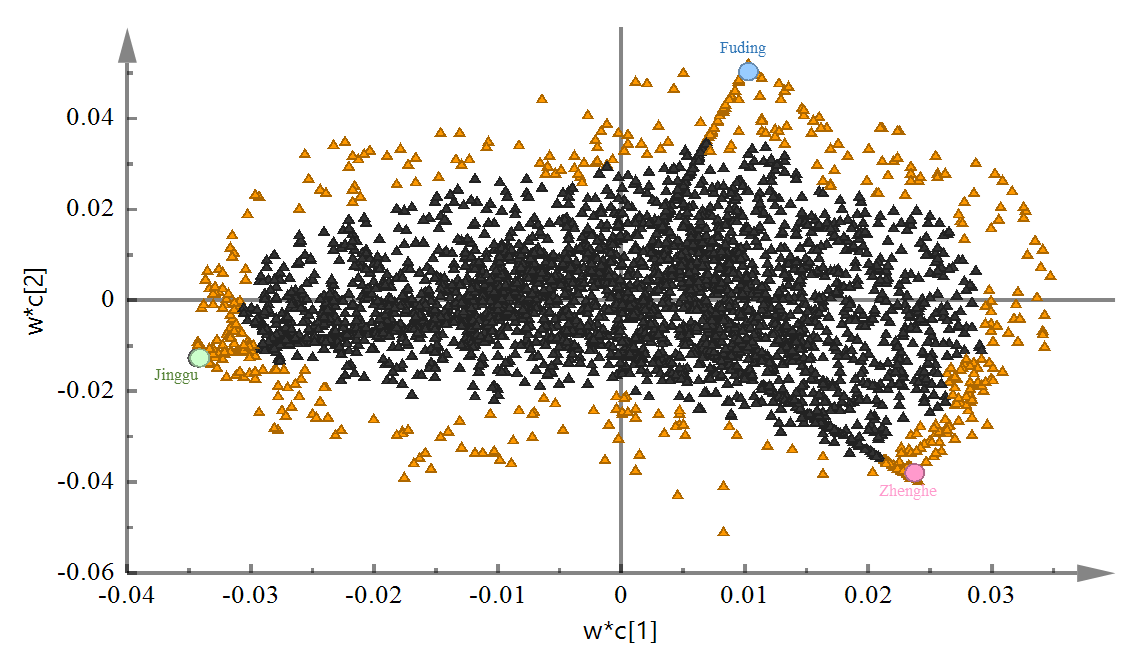


Figure S3. Loading plot for differential compounds among Zhenghe, Fuding and Jinggu white tea samples.


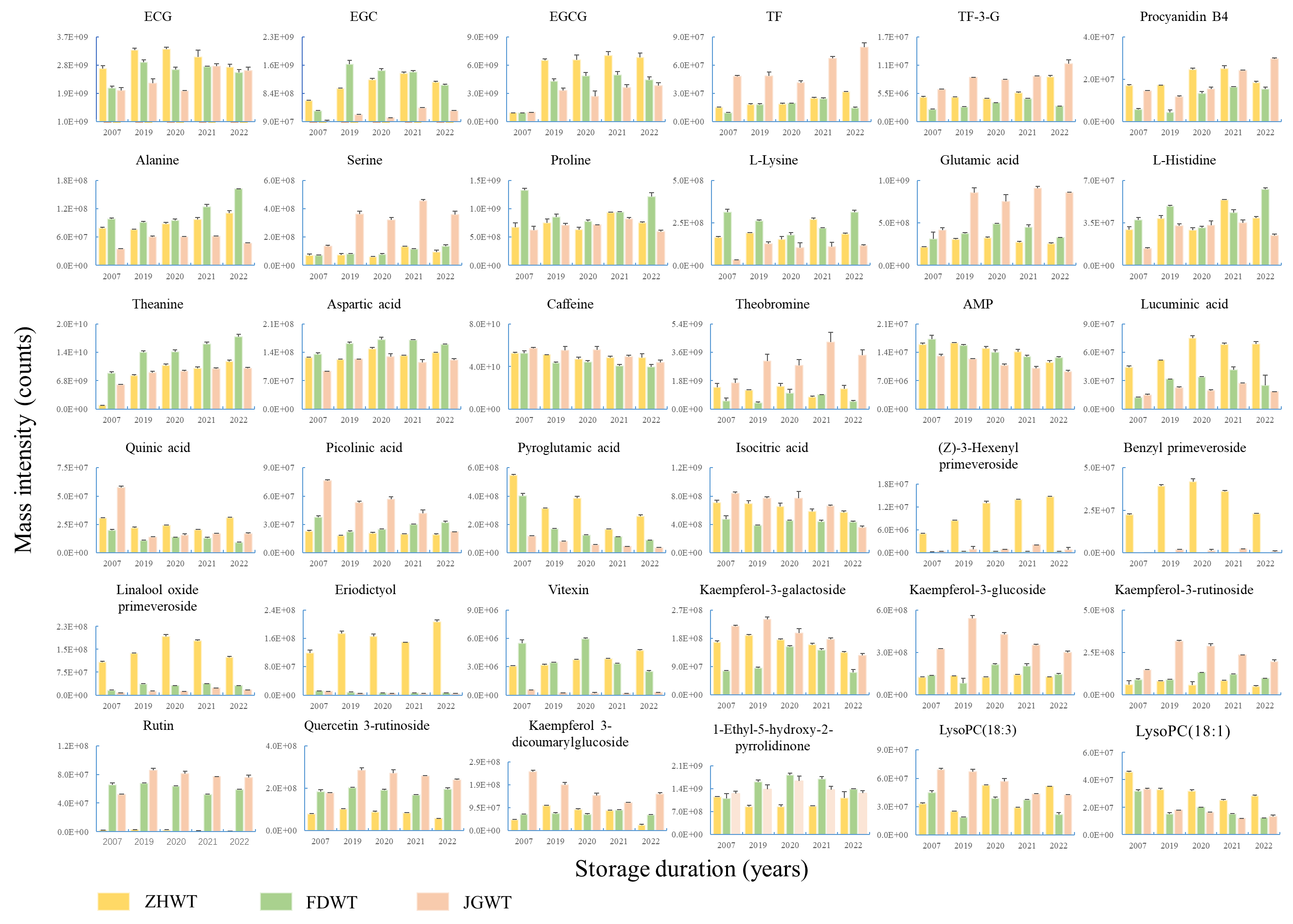


Figure S4. Typical change patterns of representative compounds during white teas storage.
